# Posterior shoulder instability – A systematic review and meta-analysis of glenoid osteotomy and bone block procedures

**DOI:** 10.1016/j.xrrt.2025.03.004

**Published:** 2025-04-02

**Authors:** Jonathan P. Evans, Samuel Handshin, Gregory Bain

**Affiliations:** a University of Exeter Medical School, Exeter, UK; b Flinders Medical Centre, Adelaide, Australia; c Flinders University, Adelaide, Australia; d Department of Orthopaedic Surgery, Flinders Medical Centre, Adelaide, Australia; e Flinders University, Adelaide, Australia

**Keywords:** Shoulder instability, Review, Meta-analysis, Glenoid osteotomy, Bone-block, Shoulder dislocation, Posterior shoulder instability

## Abstract

**Background:**

Osseous glenoid surgery for posterior shoulder instability is a low-volume, high-complexity procedure. Consequently, evidence is limited to small case series from a specialist center. This study aims to systematically review and meta-analyze the effectiveness and complications profile of glenoid osteotomy and bone block procedures in treating posterior shoulder instability.

**Methods:**

A systematic search in MEDLINE and Embase identified studies involving adult patients undergoing bone block or glenoid osteotomy. Two reviewers screened and extracted data, including patient demographics, surgery types, recurrence rates, complications, and patient-reported outcomes. The methodological quality of each study was evaluated. Meta-analysis was conducted on the reported proportion of recurrence and complications.

**Results:**

Twenty-four studies met the inclusion criteria, with over 300 patients in both osteotomy and bone block groups. The osteotomy group was more predominantly male, younger, with increased retroversion and bilateral symptoms. Rates of symptomatic recurrence were 18% (95% CI: 4, 19) for glenoid osteotomy and 11% (8, 31) for bone block group. Postoperative degenerative change was found in 5% (0, 16) and 8% (1, 18), respectively. Fracture rate was 2% (0, 8) for the osteotomy, and graft lysis rate was 12% (1, 30) for the bone block. Patient-reported outcome reporting was highly heterogenous and could not be synthesized.

**Conclusion:**

Both glenoid osteotomy and bone block procedures can successfully address symptomatic posterior shoulder instability. However, due to the considerable recurrence and complication profile, careful patient selection and consideration of centralized service delivery is warranted. The study underscores the need for further research in developing specialized, patient-centric interventions and emphasizes the importance of comprehensive outcome assessments for effective management of posterior shoulder instability.

Recurrent posterior glenohumeral instability is an uncommon but complex clinical entity. The condition is characterized by a spectrum of dynamic and/or static posterior displacement of the humeral head on the face of the glenoid. In “dynamic” posterior instability, the humeral head translates posteriorly when the arm is elevated and internally rotated, which worsens with axial loading.^4^ This tends to present in adolescents and may be associated with trauma. In the “static” forms, the humeral head is permanently decentered in the resting position and characteristically presents in older patients and is considered to be a prearthritic condition.^42^

Nonoperative treatment is typically trialed first. Woodmass et al^47^ reported that 70% of patients with new-onset posterior instability underwent surgery within 10 years of diagnosis, and in those treated nonoperatively, over 50% continued to have posterior shoulder pain.^29^ Soft tissue procedures, including capsulolabral repair or capsulorraphy, have variable outcomes, with studies in tertiary referral hospitals reporting up to a 53% revision rate at 35 months.^1^ Comparative analysis of the outcomes of arthroscopic Bankart repair for anterior or posterior instability found statistically worse functional scores and return to sport in the posterior group.^41^ Biomechanical analysis has demonstrated that capsulolabral repair in shoulders with >10° glenoid retroversion is unlikely to restore stability.^30^ Provencher et al^2^ has indicated that a threshold of >11% of posterior glenoid bone loss is associated with a 10-fold increase in capsulolabral repair failure.

Recent image-matching studies have defined the 3D morphological differences between patients with posterior shoulder instability and healthy control (1). The osseous changes include increased glenoid retroversion, an acromion that is shorter posterolaterally, higher and more horizontal,^4^ and a narrower glenoid with a deficient posterior osseous rim.^45^ Furthermore, the muscular volume of the deltoid and trapezius are significantly reduced.^14^

Because of the increasingly comprehensive understanding of the osteology (1, 3), the poor outcome of rehabilitation, and soft tissue reconstructive surgery (5, 7), greater attention has been placed on directed osseous reconstructive surgery for posterior shoulder instability. Procedures, such as glenoid osteotomies and bone block procedures are being recommended for revision cases, structural bony abnormalities, or deficient posterior soft tissue.^2^^,^^13^ However, case series and systematic reviews have reported high complication and failure rates with glenoid osteotomy and bone block procedures.^9^ Meta-analysis of recurrence rates and complication profiles of these two treatment approaches has not previously been conducted.

This study aims to assess the treatment effect and complication profile of glenoid osseous procedures for the treatment of posterior shoulder instability. This includes glenoid osteotomy and bone block procedures. This information is of relevance to surgeons and patients alike and informs treatment decisions and discussions surrounding consent and risk profiling.

## Methods

### Search strategy

We performed a systematic review according to the Preferred Reporting Items for Systematic reviews and Meta-Analyses guidelines.^35^ A literature search was conducted on the second of March 2023 using MEDLINE and Embase. Articles were identified with the use of electronic search “multipurpose” field (.mp) search terms identifying posterior shoulder instability articles (Supplementary Appendix S1).

### Eligibility criteria

Studies were included if they matched all the following criteria: (1) English Language; (2) adult (>18 years) patients undergoing bone block or glenoid osteotomy for posterior shoulder instability; (3) randomized trials, cohort, or case series; and (4) reporting complications. The following types of studies were excluded: posterior soft tissue repair or reconstruction procedures, review articles, cadaveric, and biomechanical studies.

### Study selection

Two authors (J.E. + S.H.) independently screened the titles and abstracts using a purposely designed systematic review program (Rayyan QCRI; Qatar Computing Research Institute, Doha, Qatar) (15). Disagreements were resolved by another author (G.B.).

### Data extraction

Data extracted included publication date, patient demographics, preoperative retroversion and % bone loss, surgery type, graft type, length of follow-up, symptom recurrence (defined as recurrence of subjective instability and/or objective clinical or radiographic instability), patient-reported outcome measures, complications, and revision surgery.

### Data evaluation

The methodological quality of each study was assessed using a nonsummative four-point system described by Wylde et al (16). This system assesses quality based on (1) consecutive cases (2) multicenter (3) less than 20% loss to follow-up, and (4) the use of multivariable analysis. This method has been reported in recent large orthopedic systematic reviews of complication profiles.

### Data synthesis and meta-analysis

Complication categories were decided upon prior to extraction. These included fracture, postoperative degenerative change, infection, neurological injury, nonunion of graft or osteotomy, graft reabsorption, graft malposition and metalwork-related complications, recurrence, and reoperation.

For the meta-analysis, the rate of complication (%) with 95% confidence intervals (CI) was the primary exposure across studies. Analysis was performed following the methodology previously described by Kunutsor et al utilizing the Metaprop package for Stata for analysis of binomial data.^34^ The rates were calculated using the Freeman–Tukey variance, stabilizing double arcine transformation (20); this was utilized, as the use of inverse variance in fixed-effects meta-analysis is suboptimal when dealing with binary data with low rates and does not exclude studies with zero rates (18). Rates were pooled using a random effects model to minimize the expected high level of between-study heterogeneity. Heterogeneity was reported as I^2^, with a threshold for considerable heterogeneity set at >50%.^10^

All statistical analysis was performed in Stata 16 (2019 Stata Statistical Software: Release 16; StataCorp, College Station, TX, USA).

## Results

**Figure 1:**
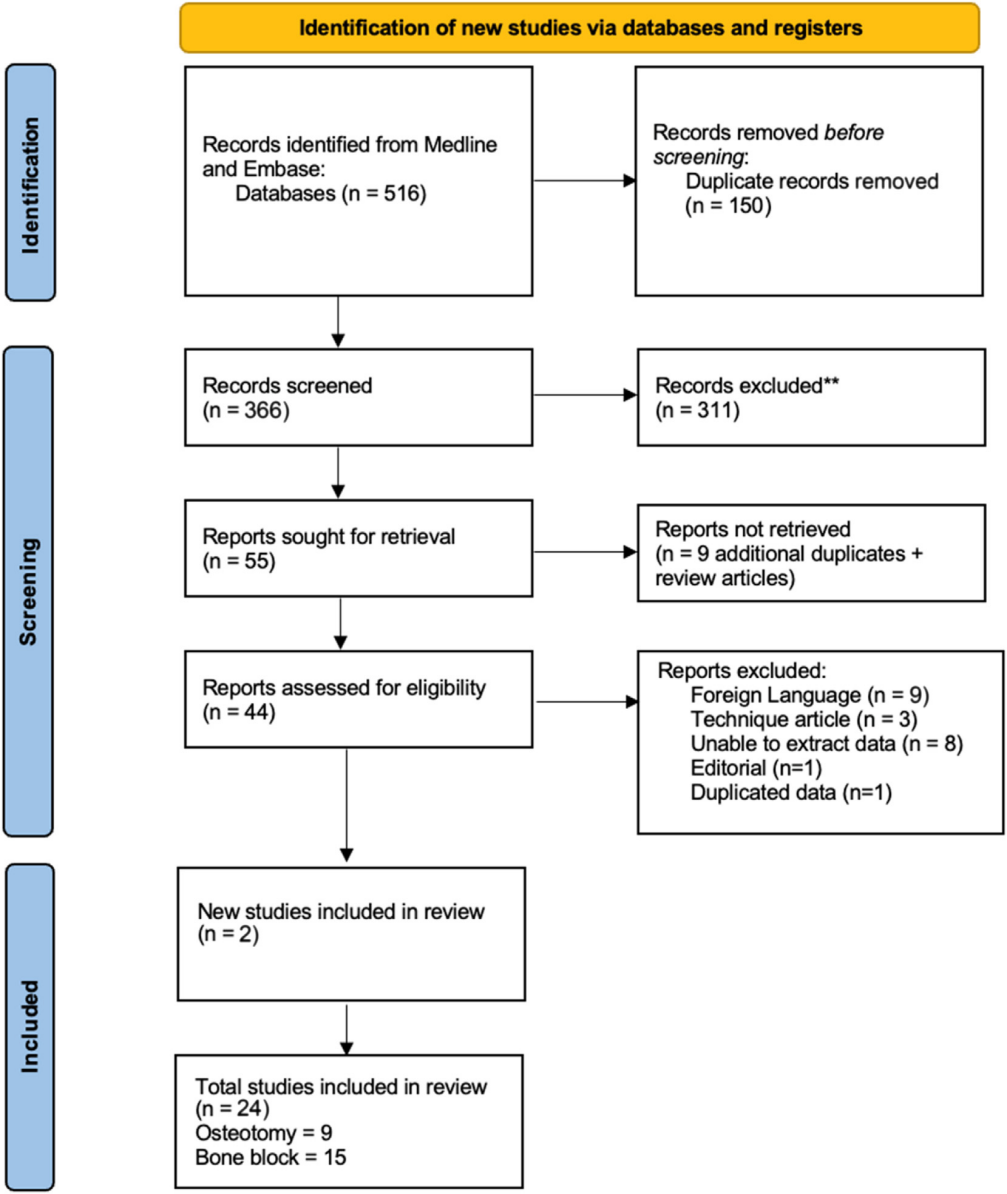
Preferred Reporting Items for Systematic reviews and Meta-Analyses flowchart of systematic review data extraction.

We identified 516 potentially relevant articles. Following assessment and exclusions, 24 studies met the inclusion criteria and underwent full-text data extraction. The results are presented in the Preferred Reporting Items for Systematic reviews and Meta-Analyses flow diagram (Fig. 1).

Over 300 patients were identified in the osteotomy and bone block groups with well-matched demographics (Table I). The mean symptom duration was only reported clearly in one of the osteotomy articles. The mean follow-up exceeded 5 years in both groups. Individual study information is presented in Table II.

**Table I:** Patient characteristics.

Characteristics	Bone block	Osteotomy
Patients	308	314
Shoulders (bilateral %)	319 (3.6%)	381 (21.3%)
Male (%)	65.3%	71%
Mean age at surgery	26.3 (SD 4.7)	22.4 (SD 4.4)
Previous shoulder instability surgery (%)	24.0%	19.1%
Preoperative retroversion	9.2° (SD 1.5°)	15.2° (SD 6.7°)
Follow-up (mo)	63.2 (SD 51.4)	74.0 (SD 49.1)

**Table II:** Study characteristics.

Study	Evidence level	Single/multicentre	No. total shoulder/patients	Mean age, yr (range) Male/female	Mean duration of symptoms, (mo)	Mean follow-up, mo (range)	No. lost to follow-up	Osteotomy/bone block	Opening wedge/graft type	Retroversion Pre-operative (°) Post-operative (°)	Glenoid bone loss (%)	No. recurrence (Mean recurrence duration, mo)	No. complications	PROM Mean Pre-operative (SD) Mean post-operative (SD)	Prior surgery (%)
Barbier *et al*, 2008,^3^	IV	Single	8/8	28.7 (23-33) 8 M, 0 F	36	34 (10-60)	0	BB	Ipsilateral ICBG	1 NR	NR	0 NR	6 Reoperation (3) MRC (3)	CS 82.5 96.3	3 (37.5)
Bessems *et al*, 1995,^5^	IV	Single	13/10	19 (15-27) 8 M, 2 F	NR	108 (12-192)	0	Osteotomy	OW + ICBG	NR NR	NR	0 NR	1 PODC (1)	ROWE NR 93.8	NR
Camenzind *et al*, 2021,^6^	IV	Single	49/49	36 (SD ± 13) 15 M, 34 F	NR	79 (±34)	32	BB	ICBG	NR NR	5	0 NR	25 Reoperation (8) PODC (8) Neurological Injury (1) MRC (8)	CS 60 (17) 84 (11)	8 (16.3)
Camenzind *et al*, 2021,^7^	IV	Single	19/18	33.9 (22-68) 16 M, 2 F	NR	88 (60-120)	0	BB	ICBG	NR NR	NR	1 NR	14 Reoperation (7) MRC (7)	CS/ROWE 63 (18)/37 (23) 80 (18) 79 (24)	9 (47.4)
Clavert *et al*, 2017,^8^	IV	Multicentre	66/66	27.8 (15-58) 55 M, 11 F	NR	44 (12-156)	0	BB	ICBG (86%) ABG (14%)	NR NR	NR	8 NR	33 GR (33)	CS 76.1 86	NR
Ernstbrunner *et al*, 2021,^13^	IV	Single	7/7	28.6 (16-45) 4 M, 3 F	NR	28 (24-36)	0	Osteotomy	ICBG	16 0	NR	4 NR	0	SSV/CPS 40/7 90/15	4 (57.1)
Gilat *et al*, 2020,^15^	IV	Single	10/10	24 (17-35) 8 M, 2 F	NR	34 (13-76)	NR	BB	DTA	NR NR	NR	1 NR	4 Reoperation (2) Other Wound Complication (2)	SF-12 (Physical) 32.5 41.8	7 (70)
Gosens *et al*, 2001,^16^	IV	Single	11/10	25.8 (16-57) 6 M, 4 F	NR	72 (43-102)	1	BB	ICBG	NR NR	NR	2 NR	1 Infection (1)	VAS/ROWE (Post-Op Only) 6.6 3.8/86	3 (27.3)
Graichen *et al*, 1999,^17^	IV	Single	32/17	NR	NR	60 (19-128)	1	Osteotomy	ICBG	9.35 4.62	NR	2 NR	0	ROWE/C-M (Post-Op only) 81% good/excellent	3 (9.3)
Haeni *et al*, 2018^18^	IV	Single	7/7	27 (22-34) 4 M, 3 F	NR	7 (4-72)	0	BB	ICBG	NR NR	NR	0 NR	4 Reoperation (3) PODC (1)	ROWE 30 62.5	5 (71.4)
Hawkins *et al*, 1996,^19^	IV	Single	12/12	27.4 (21-39) 11 M, 1 F	NR	61 (32-100)	1	Osteotomy	ABG (42%) ICBG (58%)	7.5 3	NR	2 NR	7 Fracture (2) PODC (1) Infection (1) Other (3) Persistent Instability (2) Coracoid Impingement (1)	VAS NR	5 (42)
Hawkins *et al*, 1984,^20^	IV	Single	26/17	16 (NR) NR	NR	86 (NR)	NR	Osteotomy	OW	NR NR	NR	2 NR	5 PODC (2) Wound complication (1) Other (2) Avascular Necrosis (1) External Rotation Contracture (1)	NR	1 (3.8)
Hernandez *et al*, 1986,^21^	IV	Single	8/8	20.4 (14-30) 5 M, 3 F	NR	36 (10-114)	NR	Osteotomy	OW	NR NR	NR	1 NR	0	NR	NR
Inui *et al*, 2002,^23^	IV	Single	249/211	20 (NR) 63 M, 148 F	NR	84 (NR)	NR	Osteotomy	OW + ICBG	NR NR	NR	35 NR	72 Reoperation (13) Fracture (7) GR (52)	ROWE 36 88	15 (6)
Lacheta *et al*, 2019,^25^	IV	Single	13/12	25 (17-40) NR	NR	19.8 (14-36)	1	Osteotomy	OW + ABG	23 13	NR	1 NR	4 Fracture (4)	ROWE/OSIS NR 90/44 Range (21-48)	4 (31)
Langlais, *et al*, 2002,^28^	IV	Single	18/18	20 (12-35) NR	NR	61.2 (NR)	0	BB	Autograft and Allograft	NR NR	NR	4 NR	7 PODC (3) GR (4)	NR	NR
Meuffels, *et al*, 2010,^32^	IV	Single	11/11	22 (14-56) 6 M, 5 F	NR	219.6 (153.6-282)	0	BB	ICBG	NR NR	NR	8 NR	15 Reoperation (4) PODC (9) GR (2)	VAS 70 25	3 (27.3)
Norwood, *et al*, 1984,^33^	IV	Single	21/21	23.1 (16-46) 18 M, 2 F	19.8	39.9 (13-73)	1	Osteotomy	OW + ABG	NR NR	NR	9 NR	0	NR	NR
Schwartz *et al*, 2013,^37^	IV	Single	19/18	29.9 (NR) 13 M, 5 F	72	20.5 (NR)	0	BB	ICBG	NR NR	NR	1 NR	9 Reoperation (7) GR (2)	ROWE 16.5 77.6	NR
Servien *et al*, 2007,^38^	IV	Single	33/33	24.8 (17-40) 19 M, 1 F	36	72 (24-228)	13	BB	ICBG	9.6 NR	NR	3 NR	6 PODC (4) Neurological Injury (1) GR (1)	CS NR 93.3 (80-103)	NR
Struck *et al*, 2016,^39^	IV	Single	15/13	20 (17-32) 7 M, 8 F	NR	46.3 (12-120)	NR	Osteotomy	SSBG (73%) ICBG (27%)	NR 12.8	NR	1 (6)	9 Reoperation (4) GR (1) MRC (4)	CS NR 82 (64-98)	NR
Villefort *et al*, 2023,^40^	IV	Single	21/21	26 (16-41) 10 M, 4 F	NR	108 (48-240)	14	BB	ABG (7%) SSBG (29%) ICBG (65%)	7.5 3.9	5	6 NR	24 Reoperation (3) PODC (9) GR (8) MRC (2) GM (2)	CS 77 (11) 83 (14)	7 (33.3)
Waltenspul *et al*, 2021,^44^	IV	Single	8/7	24 (19-34) 6 M, 1 F	NR	180 (120-228)	6	Osteotomy	OW + ICBG	20 3	NR	6 (84)	15 Reoperation (3) Fracture (2) PODC (7) MRC (1) GM (1) Posterior Labral Repair (1)	CS 70 (8) 68 (23)	2 (25)
Wellmann *et al*, 2018,^46^	IV	Single	24/18	22 (15-48) NR	NR	26.4 (14-44)	0	BB	ICBG	10.5 NR	NR	3 NR	43 PODC (3) GR (22) MRC (16) GM (1) Other (1) Pneumothorax	ROWE 50 (37-62) 75 (40-95)	NR

*ABG*, acromial bone graft; *BB*, bone block; *C-M*, Constant-Murley comparison; *CPS*, Constant pain score; *CS*, Constant score; *DTA*, distal tibial allograft; *GM*, graft malposition; *GR*, graft reabsorption; *ICBG*, iliac crest bone graft; *MRC*, metalwork-related complication; *NR*, not reported; *OSIS*, Oxford Shoulder Instability Score; *OW*, opening wedge; *PODC*, post-operative degenerative change; *PROM*, patient reported outcome measure; *ROWE*, Rowe score; *SSBG*, scapular spine bone graft; *SSV*, subjective shoulder value; *VAS*, visual analog scale.

All osteotomies were opening wedge through a posterior approach. In six articles, iliac crest bone graft was used, and in two articles, acromial graft was used. The mean preoperative retroversion of 15.2° (SD 6.7°) was corrected to 4.7° (SD 4.9°). In the bone block group, iliac crest graft was used in 13 articles, scapular spine graft in 2, and distal tibial allograft in 1 article. The mean preoperative retroversion was 9.2° (SD 1.5°)

Patient-reported outcomes were poorly reported throughout. The Constant score was found to be the most widely reported outcome measure. However, this outcome has been reported as ineffective in the evaluation of shoulder instability and has not been validated for assessing improvements in shoulder function after instability surgery.^11^ More than 50% of studies only reported postoperative scores. Owing to the data attrition and heterogeneity of measures used, a formal meta-analysis was not performed on the patient-reported outcomes. Of the five bone block studies that reported preoperative and postoperative scores, the mean improvement in the Constant score was 7.1. Disparate outcome utilization impeded any assessment of average improvement in the osteotomy group.

**Figure 2:**
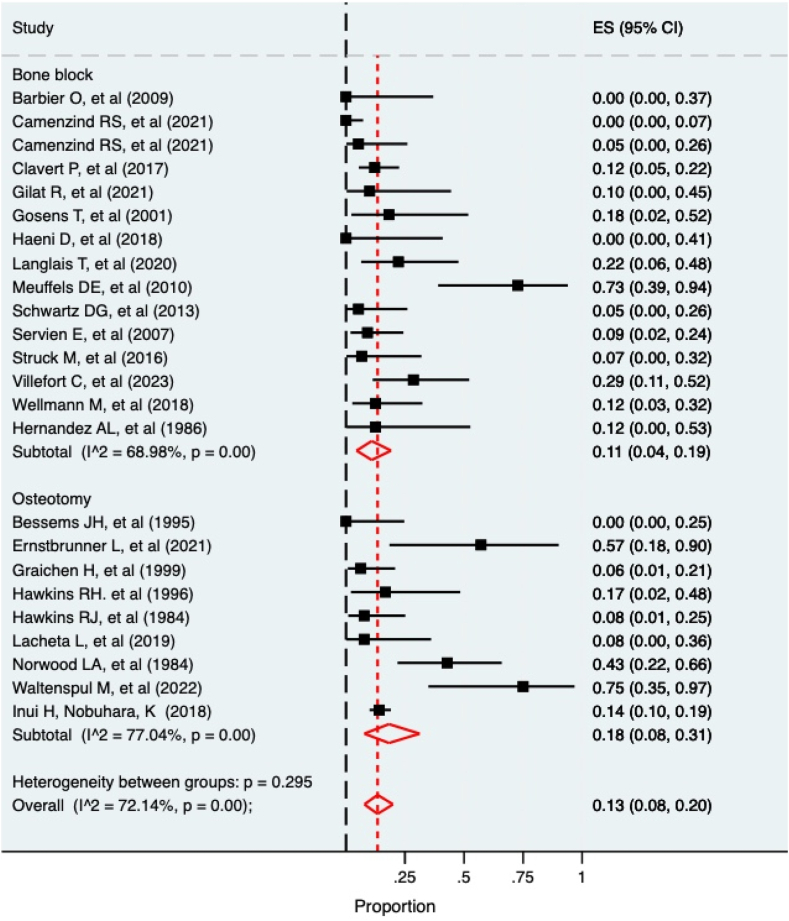
Forest plot of recurrence rate.

**Figure 3:**
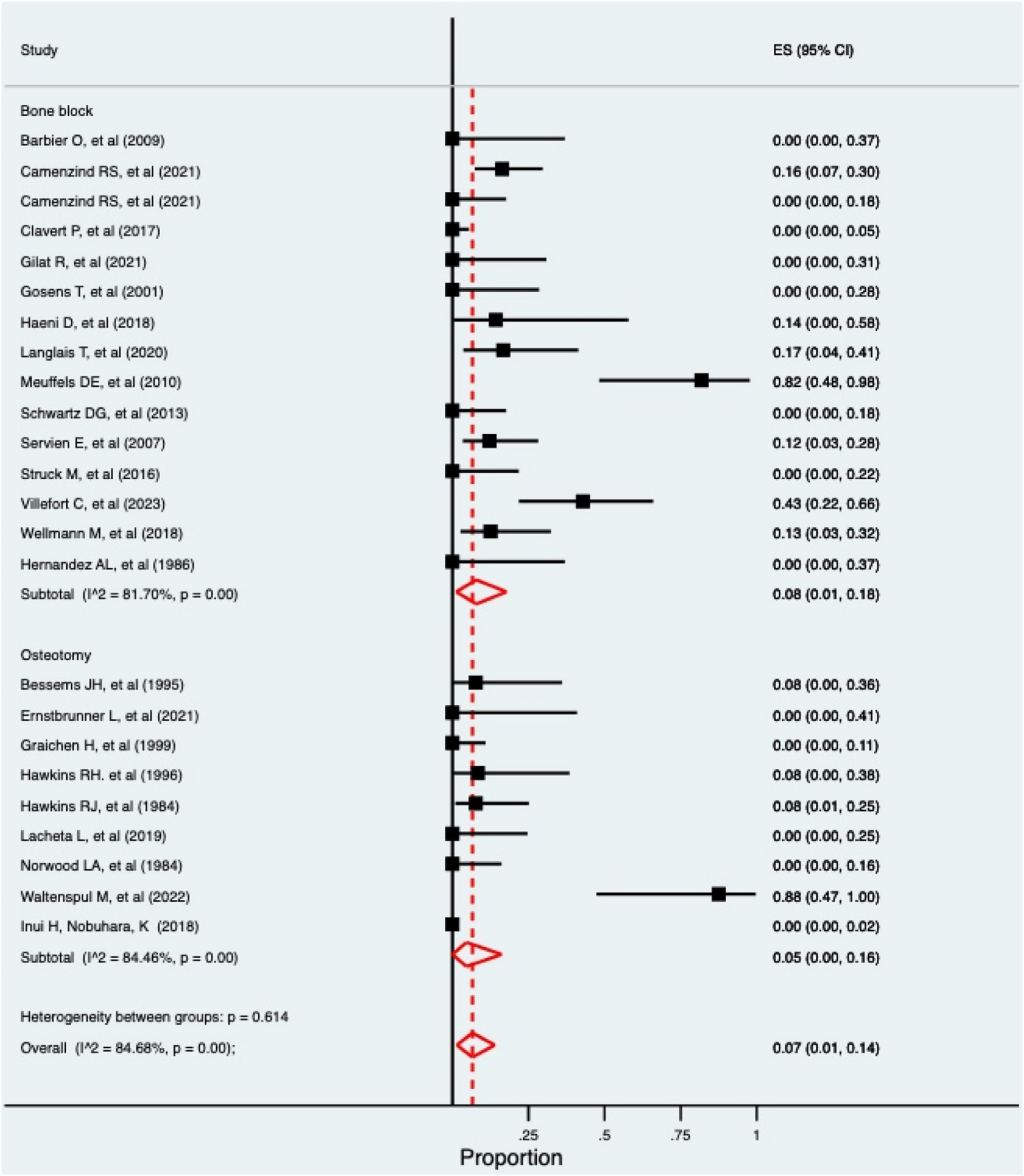
Forest plot of postoperative degenerative change.

The results of the meta-analysis are presented in Table III, with the recurrence meta-analysis in Figure 2 and postoperative degenerative change in Figure 3. High levels of within-group and between-group heterogeneity was observed with I^2^ consistently over 50%. This likely reflects the methodological diversity and small sample sizes. Of the nonrecurrence complications, a cumulative 252 instances were recorded from 700 cases (36%). Of these, 52 of 319 (16.3%) cases were in the osteotomy group, and 200 of 381 (52%) cases were in the bone block group. Of the complications not recorded in the meta-analysis, 1 infection was recorded in each group, two neurological injuries were reported in the bone block group, and 1 pneumothorax was reported in the bone block group. No studies reported any episodes of non or malunion in either the bone block or osteotomy groups. A single episode of graft mal position was reported in 1 bone block study^46^ and 1 osteotomy study.^43^

**Table III:** Meta-analysis results of complication risk (95% CIs).

Complication	Bone block	Osteotomy
Fracture	0%	2% (0, 8%)
Graft lysis	12% (1, 30%)	-
Recurrence	11% (4, 19%)	18% (8, 31%)
Reoperation	12% (3, 22%)	1% (0, 4%)
Postop degenerative change	8% (1, 18%)	5% (0, 16%)

Quality assessment of the bone block studies identified that 10 (66%) were consecutive series, 1 was a multicenter study (7%), and 12 (80%) had more than 80% follow-up (mean loss to follow-up 9.8%, range 0%-65%). Within the glenoid osteotomy studies, 8 (88%) were consecutive, none were multicenter, none had less than 80% follow-up (mean loss to follow-up 4%, range 0%-12.5%). No studies in either group performed a multivariant analysis of their results.

## Discussion

This study is the first meta-analysis of the symptomatic recurrence rate and complication profile of glenoid osteotomy and bone block procedures in posterior shoulder instability. We have identified that the recurrence of symptoms was 18% in glenoid osteotomy and 11% in the bone block group. For both procedures, high rates of complications were observed.

The osteotomy patients were a more complex group, they were more likely to have bilateral symptoms (21.3% vs. 3.6%), with greater glenoid retroversion (15.2° vs. 9.2°) (Table I). There was a higher proportion of male patients (71% vs. 65.3%), and they had their surgery at a younger age (22.4 vs. 26.3 years), with a shorter period of symptoms (19.8 vs. 48 months). The indication for the glenoid osteotomy has been more focused toward the dysplastic retroverted glenoid. Therefore, they are more likely to be associated to muscular^14^ and osseous changes, narrow glenoid, and acromial dysplasia^4^^,^^45^ of the shoulder. In addition, it is well recognized in the anterior shoulder instability literature that the younger males requiring surgery are often involved in high-contact sports, and that these cohorts have a poorer prognosis with nonoperative and operative treatment.^12^^,^^41^

In this review, a pragmatic approach was taken to the reporting of recurrence. Within the data extraction, episodes of recurrence included true dislocation, subluxation, or continued patient-reported symptomatic instances of instability. This approach was taken to capture the recurrence of what a patient would deem as relevant and to provide the best estimate of treatment success. This approach is, of course, not without limitations due to the heterogeneity of reporting. Without a more uniform use of patient-reported outcome measures that adequately quantify symptomatic instability, the authors feel it forms the best available estimate.

This study has identified that the bone block procedures had less recurrent instability (11% (95% CI: 4%, 19%)) compared to the glenoid osteotomy group (18% (95% CI: 8%, 31%)). It is important to note the wide CIs, that do cross, but a disparity between the groups is evident. The groups were different regarding their preoperative retroversion, with the osteotomy group being six of the key abnormalities in glenoid degrees greater (15°) in comparison with the bone block group (9°). It is plausible that their preoperative symptom profile was therefore different, and direct comparisons should come with caution. However, the mean correction placed this group back to 4° of retroversion, and instability symptoms persisted. As noted by Waltenspul et al,^43^ even in the context of correction, static posterior subluxation has been noted to continue and may contribute to the persistence of symptoms.

The bone block procedures, although not directly correcting retroversion, increase the width of the congruent arc of the glenoid face. In concert with glenoid retroversion, a morphologically narrow glenoid with a deficiency of the posterior osseous rim is commonly observed in atraumatic posterior instability,^45^ but as yet, the relative contribution of these two bony abnormalities is unknown. In cases along this spectrum of glenoid dysplasia, neither the glenoid osteotomy nor bone block procedures adequately address all the morphological elements, but results from this evidence synthesis may suggest that the increased excursion distance created by the bone block may contribute more to the reduction in instability symptoms. The technique described by Gerber, in which osteotomy is undertaken in combination with a J-shaped graft that increases the width of the glenoid, thereby addressing two of the key abnormalities in glenoid morphology, has recently been reported.^13^ Further evaluation is required; however, early reports continue to demonstrate posterior apprehension in four out of seven were prior to 2000 patients. It is also relevant to note that neither of these procedures correct or modify other bony morphological abnormalities found in posterior instability, including high acromial tilt^4^ and glenoid concavity.^23^ Recent biomechanical studies have found that posterior translation was only corrected when glenoid and acromial anatomy was restored; however, case series of these techniques have not yet been published.^22^

Within this review, it was also identified that the highest levels of recurrence were seen with the longest duration of follow-up. Meuffels et al (2010)^32^ in the bone block group and Waltenspul et al (2022)^43^ in the osteotomy group, followed their patients for 18 and 15 years, respectively, and both reported recurrence of over 70%. This very high recurrence rate at extended follow-up is of concern in a group undergoing surgery at a relatively young age. It may highlight the importance of performing corrective ostomies well before early degenerative changes occur, especially in the group with significant glenoid retroversion. In this special group, it is well-known that the natural history and the results of soft tissue repair and reconstruction are poor.^1^^,^^29^^,^^47^ The natural approach when these patients present is to trial nonoperative treatment, followed by a soft tissue procedure. However, this delays the osseous reconstructive procedure, which corrects the deformity and may reduce the advancement of degeneration.

Postoperative degenerative change was reported in 8% and 5% of cases for bone block and osteotomy groups, respectively. In concordance with the recurrence findings, studies with longer follow-up reported the highest rates. Both Meuffels et al^32^ (bone block) and Waltenspul et al^43^ (osteotomy) reported rates over 80%. This is highly relevant in a population that even after 15 years of follow-up, may well be under the age of 40, and further management conflicts with concerns regarding arthroplasty longevity. The reporting of degenerative change was highly variable, with limited use of standardized classification systems, such as the Samilson and Prieto or Kellgren and Lawrence classifications^24^^,^^36^; therefore, the results should be considered with caution. The reported rate of new or progressive degenerative change is less than recent reports for anterior instability surgery^27^^,^^31^ and may be an underrepresentation. Five of the nine osteotomy publications were prior to 2000, and as such, the assessment was often with plane radiographs rather than computed tomography, which would underestimate early degenerative changes. Due to disparate reporting within the studies, no clear trend between delayed diagnosis or duration of symptoms and the risk of developing degenerative changes could be elucidated.

Reoperation following surgery was reported to be higher in the bone block group (12% (95% CI: 3, 22)) compared to the osteotomy group (1% (95% CI: 0, 4)). Most cases of reoperation were metalwork removal due to posterior screw irritation. The possible need for metalwork removal should be communicated to the patients; however, the rate of metalwork removal is similar to that reported in anterior stabilization surgery.^26^ In both bone block and osteotomy, the procedure represents the final reconstructive surgical option, and the need for a conversion to a salvage procedure was very low.^27^^,^^31^^,^^43^

The cumulative incidence of complications was found to be higher in the osteotomy group, which is a technically demanding procedure, with reports published almost exclusively from tertiary referral center. A major concern is the propagation of the osteotomy and glenoid fracture. Within this meta-analysis, it was 2% (95% CI: 0, 8). However, individual reports were as high as 31%, and three of the 10 series estimated their rate to be 17% or higher,^19^^,^^25^^,^^43^ which reflects a large inter-study discrepancy in reporting either high rates or absence of fracture. Of note, Lacheta et al (2019) and Hawkins et al (1996) both assessed fracture propagation based on postoperative computed tomography assessment, which was atypical for the whole study sample.^19^^,^^25^

This review highlights the need for posterior shoulder instability management to continue to evolve, and the requirement for more rigorous outcome reporting. Surgically, as best as we can infer from the reported case series, the outcome of bone block and glenoid osteotomy is variable in many instances. The increasing understanding surrounding the complex interplay between the bony morphological and capsulolabral deficits that impart posterior instability will inform the newest iteration of surgical techniques. It is likely that this will become more individually focused on patient-specific deficits and may harness emerging technologies, including advanced 3D planning, customized cutting jigs, and robotic adjuncts. Controlling posterior translation may benefit from a focus on dynamic soft tissue restraint as well as bony correction in a partially analogous fashion to anterior instability surgery. Currently, many techniques are siloed in a specialist center, although there is an argument that, to benefit the widest pool of patients, efforts should be made to improve the reliability and translatability to a wider body of shoulder surgeons. If this is not feasible, a clear argument for centralization of services is needed. Importantly, future investigations should rigorously report validated outcomes, and the community of investigators would benefit from the application of a core outcome set for posterior instability. Ideally, multicenter investigation and ultimately randomized trials will best inform the application of evidence-based treatment approaches, but we recognize that undertaking these large-scale studies remain a challenge in low-volume, high-complexity surgery.

There are limitations inherent to reviews of case series of uncommon procedures. The reporting heterogeneity most likely manifests as an under-representation of complication profiles within the meta-analysis. Furthermore, the variability in patient-reported outcome reporting resulted in the inability to perform an evidence synthesis; this information, in a challenging population, would be relevant and of significant utility to patients and clinicians alike. We expect there are differences and biases in the patient selection, outcomes, and complications for these two procedures. Within the meta-analysis, it should also be noted that high heterogeneity was encountered, suggesting that the studies included in the analysis differ significantly in their results. There are no randomized trials that have compared these two procedures. Rather this study represents an attempt to quantify the risk profile of these individual procedures that are employed only having failed first and sometimes second line treatments.

## Conclusion

Both glenoid osteotomy and bone block procedures may be appropriate for the treatment of symptomatic posterior shoulder instability; however, clinicians should be stringent in their patient selection and current evidence points to the surgery being the preserve of specialist center. This meta-analysis contributes the best available synthesis of evidence on the considerable recurrence rate and complication profiles, this is of utility to the surgeon and should be communicated to each patient as part of the shared decision-making process. Further study is required to assess novel surgical interventions for this challenging condition, in which there should be an emphasis on high-quality multicenter randomized trials and the collection of patient-reported, clinically relevant outcomes.

## Disclaimers:

Funding: No funding was disclosed by the authors.

Conflicts of interest: The authors, their immediate families, and any research foundation with which they are affiliated have not received any financial payments or other benefits from any commercial entity related to the subject of this article.

Open Access: For the purpose of open access, the author has applied a Creative Commons Attribution (CC BY) licence to any Author Accepted Manuscript version arising from this submission.
