# Supplementary material for: Posterior shoulder instability – A systematic review and meta-analysis of glenoid osteotomy and bone block procedures
Source: JSES Rev Rep Tech. 2025 Apr 2;5(3):506–15. doi: 10.1016/j.xrrt.2025.03.004 (PMC12277721; doi:10.1016/j.xrrt.2025.03.004)
Supplement: Supplementary Appendix S1 [file mmc1.docx]

Appendix 1. Search strategy. Run on Ovid Silver Platter on the 2^nd^ March 2023. Medline and Embase search of full database from inception to run date.

| 1 | posterior.mp. [mp=ti, ab, hw, tn, ot, dm, mf, dv, kf, fx, dq, bt, nm, ox, px, rx, an, ui, sy, ux, mx] |
| --- | --- |
| 2 | (instability or laxity or subluxation).mp. [mp=ti, ab, hw, tn, ot, dm, mf, dv, kf, fx, dq, bt, nm, ox, px, rx, an, ui, sy, ux, mx] |
| 3 | (shoulder or glenoid or glenohumeral or labrum).mp. [mp=ti, ab, hw, tn, ot, dm, mf, dv, kf, fx, dq, bt, nm, ox, px, rx, an, ui, sy, ux, mx] |
| 4 | 1 and 2 and 3 |
| 5 | (osteotomy or glenoplasty).mp. [mp=ti, ab, hw, tn, ot, dm, mf, dv, kf, fx, dq, bt, nm, ox, px, rx, an, ui, sy, ux, mx] |
| 6 | 4 and 5 |
| 7 | ((bone adj3 block$) or (bone adj3 graft$)).mp. [mp=ti, ab, hw, tn, ot, dm, mf, dv, kf, fx, dq, bt, nm, ox, px, rx, an, ui, sy, ux, mx] |
| 8 | 4 and 7 |
| 9 | 6 or 8 |
